# New Drugs, Appliances, and Things Medical

**Published:** 1889-12-07

**Authors:** 


### NEW DRUGS, APPLIANCES AND THINGS MEDICAL.

[All preparations, appliances, novelties, etc., of which a notice is desired, should be sent to The Editor, The Lodge, Porchester Square, W.]

### DAHL'S PURE MILK.

We have examined this and find it to be a useful preparation. The point of interest in its preparation is the adoption of the laboratory method of sterilisation—viz., the repeated heatings to a point considerably below boiling, leaving a period of rest between the heatings. This method has been shown not only to destroy germs, but by destroying the successive crops of germs and spores, finally to render the fluid quite free from bacterial influence. This milk has the chemical characteristics of ordinary good milk. On opening the tin the cream is found firmly risen. In fact, owing to the method of manufacture, the product is in the condition known to dairies as "welled" milk. The readmixture of this firm cream is the chief difficulty in its use, but this can be done with a little care. We found slightly warming the tin before opening (it being a cold day) and the use of an egg whisk succeed very well. We have tried the milk with coffee and in ordinary rice pudding, the two severest culinary tests one can apply: it passed muster successfully. For infants, invalids, and for yachting or colonial purposes, in fact, wherever pure fresh milk is not to be obtained, we think this one of the best preparations in the market.

